# Micro-RNAs secreted through astrocyte-derived extracellular vesicles cause neuronal network degeneration in C9orf72 ALS

**DOI:** 10.1016/j.ebiom.2018.11.067

**Published:** 2019-01-31

**Authors:** André Varcianna, Monika A. Myszczynska, Lydia M. Castelli, Brendan O'Neill, Yeseul Kim, Jordan Talbot, Sophie Nyberg, Immanuelle Nyamali, Paul R. Heath, Matthew J. Stopford, Guillaume M. Hautbergue, Laura Ferraiuolo

**Affiliations:** Sheffield Institute of Translational Neuroscience (SITraN), University of Sheffield, 385A Glossop Road, Sheffield S10 2HQ, UK

**Keywords:** Astrocytes, Neurodegeneration, Gene therapy, Axonal growth, Extracelular vesicles, miRNA, Amyotrophic lateral sclerosis

## Abstract

**Background:**

Astrocytes regulate neuronal function, synaptic formation and maintenance partly through secreted extracellular vesicles (EVs). In amyotrophic lateral sclerosis (ALS) astrocytes display a toxic phenotype that contributes to motor neuron (MN) degeneration.

**Methods:**

We used human induced astrocytes (iAstrocytes) from 3 ALS patients carrying C9orf72 mutations and 3 non-affected donors to investigate the role of astrocyte-derived EVs (ADEVs) in ALS astrocyte toxicity. ADEVs were isolated from iAstrocyte conditioned medium *via* ultracentrifugation and resuspended in fresh astrocyte medium before testing ADEV impact on HB9-GFP^+^ mouse motor neurons (Hb9-GFP^+^ MN). We used post-mortem brain and spinal cord tissue from 3 sporadic ALS and 3 non-ALS cases for PCR analysis.

**Findings:**

We report that EV formation and miRNA cargo are dysregulated in C9ORF72-ALS iAstrocytes and this affects neurite network maintenance and MN survival *in vitro*. In particular, we have identified downregulation of miR-494-3p, a negative regulator of semaphorin 3A (SEMA3A) and other targets involved in axonal maintenance. We show here that by restoring miR-494-3p levels through expression of an engineered miRNA mimic we can downregulate Sema3A levels in MNs and increases MN survival *in vitro*. Consistently, we also report lower levels of mir-494-3p in cortico-spinal tract tissue isolated from sporadic ALS donors, thus supporting the pathological importance of this pathway in MNs and its therapeutic potential.

**Interpretation:**

ALS ADEVs and their miRNA cargo are involved in MN death in ALS and we have identified miR-494-3p as a potential therapeutic target.

Funding: Thierry Latran Fondation and Academy of Medical Sciences.

**Research in context**

**Evidence before this study**

Amyotrophic lateral sclerosis (ALS) is a neurodegenerative disease characterized by motor neuron death, however, astrocytes contribute to the neurodegenerative process. Recent studies have demonstrated that astrocytes from ALS patients can induce motor neuron death through secreted factors. Several studies have focused on secreted proteins without identifying a culprit, however.

Recent studies have highlighted the importance of microRNAs in cell-to-cell communication and have implicated miRNAs in the cross-talk between motor neurons and astrocytes in ALS.

**Added value of this study**

In this study we tested the effect of extracellular vesicles (EVs) secreted by astrocytes from ALS patients as opposed to non-ALS donors on motor neuron survival. We found that EVs secreted by ALS astrocytes induce motor neuron death nearly at the same levels as the conditioned medium. Moreover, we found that the main component of EVs are miRNAs, molecules that negatively regulate gene expression. Through a microarray study we identified several dysregulated miRNA that are likely to disrupt neuronal function once taken up by motor neurons. In particular, we focused on miR-494-3p that downregulates various genes including semaphorin3A, which is involved in axonal growth and maintenance.

**Implications of all the available evidence**

This study has uncovered the functional importance of EV secretion dysregulation that had been previously described in C9ORF72-ALS samples. Through the use of patient-derived astrocytes we have identified a number of new potential therapeutic targets for ALS that can be manipulated to restore neuronal function and prevent motor neuron death. Although challenging to use in gene therapy due to their ability to target several transcripts at once, our study provides evidence that manipulation of individual miRNAs can lead to significant beneficial downstream effects *in vitro* to be validated *in vivo*.

Alt-text: Unlabelled Box

## 1 Introduction

Amyotrophic lateral sclerosis (ALS) is a fatal neurodegenerative disease characterized by motor neuron (MN) degeneration. The mechanisms and sequence of events leading to MN death are still widely unknown, but the observation that the first pathophysiological changes observed in patients involve neuromuscular junction (NMJ) disruption have given rise to the theory known as the ‘dying-back’ hypothesis [1]. Dysregulated RNA metabolism is strongly implicated in this pathogenesis [2], as demonstrated by the many ALS-linked genes encoding proteins involved in RNA metabolism, such as *TARDBP* and *FUS* [3]. In particular, microRNA (miRNA) metabolism dysregulation has also been implicated in ALS [[4], [5], [6]].

In addition, polymorphic (G4C2)n hexanucleotide repeat expansions within the *C9ORF72* gene are the most common genetic cause of ALS and frontotemporal dementia [7,8]. They are known to mediate neurotoxicity through multiple mechanisms including alterations of pre-mRNA processing [9,10], along with dysregulations of autophagy, protein homeostasis and vesicle trafficking [[11], [12], [13]].

Although ALS is characterized by MN degeneration, rodent studies have demonstrated that astrocytes dictate disease progression *in vivo* [14]and patient-derived astrocytes are toxic towards wild-type MNs through both cell-to-cell contact and secreted factors *in vitro* [[15], [16], [17]]. While several hypotheses have been put forward [18,19], there is no consensus on the nature of these toxic factors.

Under normal physiological conditions, astrocytes regulate many neuronal functions including axon maintenance [20], and at least part of this communication is regulated through secreted extracellular vesicles (EVs) [21,22]. Specifically, EV miRNA cargo can modulate neuronal and astrocytic function in health and disease [23,24]. In the context of ALS, recent rodent-based studies have implicated cell-secreted miRNA signaling in a number of pathogenic processes, from excitotoxicity [24] to neuromuscular junction disruption [25].

Based on our previous research, we sought to determine whether astrocyte-derived extracellular vesicles (ADEV) from ALS patients contain distinct and/or altered levels of miRNAs that would account for the astrocyte toxicity reported against MNs [16].

Here we show that astrocytes derived from C9ORF72-ALS patients have impaired EV formation. We also show that the miRNA cargo of these EVs is specific to astrocytes compared to the fibroblasts of origin and significantly differs from healthy control astrocytes. Specifically, we have identified miR-494-3p as a key regulator of Semaphorin 3A (Sema3A) and other molecules involved in axonal maintenance, with dramatic consequences on axonal/neurite length and motor neuron survival *in vitro*. Mir-494-3p dysregulation was also detected in the cortico-spinal tract isolated from post-mortem ALS biopsies, thus corroborating the importance of this pathway in disease and supporting its validity for future therapeutic development.

## 2 Materials and methods

### 2.1 Human sample ethics statement

All skin biopsy donors (Table 1) provided informed consent before sample collection (University of Sheffield, Study number STH16573, Research Committee reference 12/YH/0330 or Coriell Institute).

**Table 1:** Details of fibroblast donors.

Cell line	Diagnosis	Mutation	Age at collection (y)	Gender	Source	Identifier
AG08620	Non-ALS	–	64	Female	Coriell Institute	RRID:CVCL_4L17
155	Non-ALS	–	42	Male	UoS	RRID:CVCL_UF81
3050	Non-ALS	–	55	Male	UoS	RRID: CVCL_UH66
78	fALS	C9ORF72	68	Male	UoS	RRID:CVCL_UF84
183	fALS	C9ORF72	51	Male	UoS	RRID:CVCL_UF85
201	fALS	C9ORF72	66	Female	UoS	RRID:CVCL_UF86

### 2.2 Conversion of skin fibroblasts to induced neural progenitor cells (iNPCs)

Skin fibroblasts from 3 controls and 3 C9-ALS patients (Table 1) were reprogrammed as previously described [16]. Briefly, 10^4^ fibroblasts were grown in one well of a six-well plate. Day one post seeding the cells were transduced with retroviral vectors containing Oct 3/4, Sox 2, Klf 4 and c-Myc. Following one day of recovery in fibroblast medium, DMEM (Gibco, Waltham, MA, USA) and 10% FBS (Life Science Production, Bedford, UK) the cells were washed 1× with PBS and the culture medium was changed to NPC conversion medium comprised of DMEM/F12 (1:1) GlutaMax (Gibco, Waltham, MA, USA), 1% N2 (Gibco, Waltham, MA, USA), 1% B27 (Gibco, Waltham, MA, USA), 20 ng/ml FGF2 (Peprotech, Rocky Hill, NJ, USA), 20 ng/ml EGF (Peprotech, Rocky Hill, NJ, USA) and 5 ng/ml heparin (Sigma, St. Louis, MO, USA). As the cell morphology changes and cells develop a sphere-like form they can be expanded into individual wells of a six-well plate. Once an iNPC culture is established, the media is switched to NPC proliferation media consisting of DMEM/F12 (1:1) GlutaMax, 1% N2, 1% B27, and 40 ng/ml FGF2.

### 2.3 iAstrocyte differentiation and maintenance

iAstrocytes were yielded as previously described [9,16]. Briefly, iNPCs were switched to astrocyte proliferation media, DMEM (Fisher Scientific, Hampton, NH, USA), 10% FBS (Life science production, Bedford, UK), 0.2% N2(Gibco, Waltham, MA, USA). Cells were grown in 10 cm dishes coated with fibronectin for 7 days unless otherwise stated.

For Nanoparticle Tracking Analysis experiments, astrocyte medium was switched to EV free medium (DMEM (Gibco, Waltham, MA, USA) and 10% (v/v) knockout serum replacement (Gibco, Waltham, MA, USA)) 24 h before medium collection (day 6 of differentiation). Pre-conditioned media was collected from the iastrocytes at day 7 and centrifuged at 300 ×*g* for 10 min, prior to evaluation using the ZetaView (Particle Metrix, Meerbusch, Germany).

### 2.4 MN monocultures with EVs from iAstrocytes

Murine Hb9-GFP^+^ MN cultures were prepared from mouse embryonic stem cells (mESC) containing a GFP gene controlled by the MN-specific promoter Hb9 (kind gift from Thomas Jessel, Columbia University, New York). Hb9-GFP mESC were maintained by culturing on primary mouse embryonic fibroblasts (Merck, Burlington, MA, USA) in mESC media (KnockOut DMEM (Gibco, Waltham, MA, USA), 15% (*v*/v) embryonic stem-cell FBS (Gibco, Waltham, MA, USA), 2 mM l-glutamine (Gibco, Waltham, MA, USA), 1% (*v*/v) nonessential amino acids (Gibco, Waltham, MA, USA) and 0.00072% (v/v) 2-mercaptoethanol (Sigma, St. Louis, MO, USA)). mESCs were then differentiated into MN-enriched cultures *via* embryoid bodies (EBs). Briefly, mESCs were lifted using trypsin, resuspended in EB medium (DMEM/F12 (Gibco, Waltham, MA, USA), 10% (v/v) knockout serum replacement (Gibco, Waltham, MA, USA), 1% N2 (Gibco, Waltham, MA, USA), 1 mM l-glutamine (Gibco, Waltham, MA, USA), 0.5% (*w*/*v*) glucose (Sigma, St. Louis, MO, USA) and 0.0016% (*v*/v) 2-mercaptoethanol (Sigma, St. Louis, MO, USA)) and seeded into non-adherent Petri dishes. EB media was replenished every day, and 2 μM retinoic acid (Sigma, St. Louis, MO, USA) and 0.5 μM smoothened agonist (Sigma, St. Louis, MO, USA) were added daily from day 2 to day 7 post-seeding to induce mESC differentiation into MNs. After 7 days of differentiation, EBs were dissociated using 200 U/ml papain (Sigma, St. Louis, MO, USA). Hb9-GFP^+^ MN were sorted using FACS Aria machine and 40,000 cells/well were plated in 96 well plates (Cellstar, Sigma, St. Louis, MO, USA) pre-coated with laminin 1:200 (Sigma, St. Louis, MO, USA) and polyornithine 1:1000 (Sigma, St. Louis, MO, USA) in PBS. MNs were cultured in 100 μl of MN media (Knockout DMEM, F12 medium, 10% Knockout Serum Replacement, 1 mM l-glutamine, 0.5% (*w*/*v*) glucose, 1% N2, 0.0016% (*v*/v) 2-mercaptoethanol, 20 ng/ml BDNF (Peprotech, Rocky Hill, NJ, USA), 40 ng/ml CNTF (Peprotech, Rocky Hill, NJ, USA) and 20 ng/ml GDNF (Peprotech, Rocky Hill, NJ, USA)) for 24 h before treatment with either complete astrocyte conditioned media or isolated extracellular vesicles (EVs) appropriately diluted in fresh iastrocyte medium (DMEM (Gibco, Waltham, MA, USA), 10% FBS (Life Science Production, Bedford, UK) and 0.2% N2(Gibco, Waltham, MA, USA)) in order to keep the same concentration of EVs present in the conditioned medium. Each treatment well, comprised one part MN media and two parts either complete astrocyte conditioned media or isolated EVs or isolated EVs diluted in complete MN medium (including growth factors BDNF/CNTF/GDNF at the concentrations specified above). MNs were imaged using an INCELL analyser 2000 (GE Healthcare, Chicago, IL, USA) 24 h after seeding (to confirm the number of cells before treatment), and then every 24 h onwards for 3 days. The number of viable MN (defined as GFP+ motor neuronal cell bodies with at least 1 axon) were analysed using the Columbus™ Data Storage and Analysis System (RRID:SCR_007149; Perkin Elmer, Waltham, MA, USA). The percentage MN survival was calculated as the number of viable motor neurons at day 3 as a percentage of the number of viable MN at day 0 pre-treatment.

As for treatment with miRNA mimics (MirVana, ThermoFisher, Waltham, MA, USA), scramble and hsa-miR-494-3p, were added to the conditioned medium or ADEVs on the day of treatment.

### 2.5 EV preparation

EVs were isolated from conditioned medium by ultracentrifugation at 100,000 ×*g* for 90 min at 4 °C using a 70ti rotor and Beckman Coulter Ultracentrifuge after initial collection and centrifugation for 10 min, 300 ×*g* at room temperature and filtration through a 0.22 μm filter to remove cell debris. The supernatant was then removed and the EV pellet resuspended in 300 μl DEPC treated PBS.

### 2.6 NTA

Nanoparticle tracking analysis was conducted using the ZetaView (RRID:SCR_016647; Particle Metrix, Meerbusch, Germany) and its respective software (RRID:SCR_016647; ZetaView 8.03.08.03). Prior to use the instrument was calibrated using polystyrene beads (100 nm). Conditioned media samples taken from human induced astrocyte cultures were loaded into the ZetaView cell. Nanoparticle tracking analysis measurements were recorded at 11 different positions and three cycles of readings were documented for each position. Following the withdrawal of any outlier positions the ZetaView software calculated the mean, median and mode sizes in addition to the concentration of particles within the sample.

### 2.7 EV transmitted electron microscopy

For immuno-gold EM, a 5 μl drop of resuspended EVs was deposited onto the grid and adsorbed for 20 min. Grids were washed in 100 μl drops of PBS 3 times, followed by blocking for 10 min in 100 μl of blocking buffer consisting of 5% horse serum in PBS. A 5 μl drop of Ms. CD63(TS63, Invitrogen; Cat# 10628D, RRID:AB_2532983; Carlsbad, CA, USA) of 20 μg/ml was incubated on the grid for 20 min, washing the grid in PBS 3 times at the end of incubation. 5 μl of anti-mouse IgG-10 nm gold (Cat#ab39619; RRID:AB_954440; Abcam, Cambridge, UK) in 5% horse serum was adsorbed onto the grid for 20 min, and washed with PBS. The immunoreaction was post-fixed with EM grade 3% glutaraldehyde/formaldehyde for 5 min, and the sample was contrasted with 2% uranyl acetate for 60 s and washed 3 times with distilled water before drying overnight. Samples were imaged with a Fei Tecnai 2000 electron microscope at 80 kV.

### 2.8 RNA isolation and quantitative RT-PCR

RNA was harvested using the RNeasy Plus mini Qiagen kit (Qiagen, Germantown, MD, USA) and total RNA was reverse transcribed using the High Capacity cDNA Reverse Transcription kit (Applied Biosystems, Foster City, CA, USA) in accordance with the manufacturer's instructions. Real-time quantitative PCR reactions were conducted using 2× SYBR Green qPCR Master Mix (Low ROX) (Bimake, Houston, TX, USA) and assays were run on a Stratagene Mx3000P™ Real Time Thermal Cycler (Agilent Technologies Ltd., Santa Clara, CA, USA). Mouse Sema3A RNA levels were detected and relative quantification calculated using the 2−∆∆CT method. The following primers were utilised: mouse Sema3A Fw 5’-TGTACTCTGGAACTGCTGCG-3′; Rv 5’-TCTCTGGGATGAGATGGGCA-3′; mouse GAPDH Fw 5’-GCTACACTGAGGACCAGGTTGTCT-3′; Rv 5’-AGCCCCGGCATCGAA-3′; human Sema3A Fw 5’–CACCATCACCCCATCAGGAC-3′; Rv 5’-CTCTGGGATGAGATGGGCAC-3′ and human RPL13A Fw 5’-CAAGCGGATGAACACCAACC-3′; Rv 5’-TTTTGTGGGGCAGCATACCT-3′.

For TaqMan qPCR 6 genes associated with EV formation were selected (Table 2) and PCR was performed using PrimeTime qPCR 5′ nuclease assays (IDT technologies, Coralville, IA, USA) as described above. Gene expression values were determined using the ddCt calculation following normalization to β-Actin expression, also evaluated using the associated PrimeTime qPCR 5′ nuclease assay.

**Table 2:** Primers and probes for TaqMan qPCR.

Primer name	Primer sequence (5′ – >3′)	Probe sequence (5′ – >3′)
ALIX-F	GAAGCACAGGTGGTGGAG	56FAM/CATGGTTCT/ZEN/TGGCGCTGGAGTTG
ALIX-R	CAGCAGGAGGACATGCAC	
TSG101-F	TTTTCCAGAGCAGAACTGAGT	56FAM/AACCTCGGC/ZEN/TACTTCTTGATCTAAACGG
TSG101-R	GAAAAAGGGTCACCAGAAACTG	
CHMP2B-F	TCGTCATCAGAACCGTCAAAG	56FAM/CAGAAGGAA/ZEN/AACATGAAAATGGAAATGACTGAAGA
CHMP2B-R	AGGCAGTTAACAAGAAGATGGA	
CHMP4B-F	GGAACATTTGGTAGAGGGACTG	56FAM/TGGCGGAAT/ZEN/TAGAAGAACTAGAACAGGAGG
CHMP4B-R	AGGGTTTGGAGAAGAGTTTGAC	
VPS4A-F	CTCTTGCCCAAAGTCCTCTG	56FAM/TTCTTCACT/ZEN/TTCAGGAGGTCGTCTGC
VPS4A-R	TCTTAGAGCCTGTGGTTTGC	
β-Actin-F	CCAGTGGTACGGCCAGA	56FAM/CCATGTACG/ZEN/TTGCTATCCAGGCTGT
β-Actin-R	GCGAGAAGATGACCCAGAT	

For miRNA analysis, total RNA was isolated using TRIzol™ Reagent (Invitrogen, Carlsbad, CA, USA) as per the manufacturer's guidelines TaqMan Small RNA Assay (20×) for hsa-miR-103 and hsa-miR-494-3p and TaqMan Universal PCR Master Mix II (2×) (both Life Technologies Ltd., Carlsbad, CA, USA) were used for real-time quantitative PCR reactions and the assays were conducted on a Stratagene Mx3000P™ Real Time Thermal Cycler (Agilent Technologies Ltd., Santa Clara, CA, USA).

### 2.9 Microarray analysis

Total RNA was extracted from EV pellets derived from the conditioned media of C9ORF72 patient and control patient induced astrocytes using the Direct-Zol RNA mini-prep (Zymo Research, Irvine, CA, USA) as per the manufacturer's instructions. RNA was labelled using the FlashTag biotin HSR RNA labelling kit (Affymetrix, Santa Clara, CA, USA) according to the manufacturer's instructions. Briefly, 2 μl of RNA Spike Control Oligos were added to 8 μl of RNA, whilst ATP mix was diluted 1:500 in 1 mM Tris. 5 μl of Master Mix was added to 10 μl RNA/Spike Control Oligos and incubated at 37 °C heat for 15 min (Poly (A)-tailing reaction). To the Poly (A)-tailed RNA 4 μl of 5× FlashTag Biotin HSR Ligation Mix and 2 μl of T4 DNA Ligase was added and following a 30 min incubation at room temperature the reaction was stopped by adding 2.5 μl of HSR Stop Solution.

Labelled RNA from each sample was then prepared for hybridisation. The hybridisation cocktail, comprised of 110.5 μl of Hybridization Master Mix and 21.5 μl of biotin-labelled sample was incubated at 99 °C for 5 min and then 45 °C for 5 min. The sample mixture was then loaded into an array and placed into a hybridization oven and incubated at 48 °C and 60 rpm for 16 to 18 h. Arrays were then washed and stained using Stain Cocktails 1/2 and scanned with the Affymetrix GeneChip Scanner 3000.

Affymetrix Expression Console was used to evaluate the hybridisation efficacy and probe set expression of the GeneChip miRNA Array 4.0. Differential miRNA expression between samples were analysed using Affymetrix Transcriptome Analysis Console for RNA species annotated within the human genome. The Affymetrix Expression Console was then used to evaluate probe set expression after normalization using the detected above background (DABG) method. DABG is a detection metric where the intensity from each perfect match probe is compared to a distribution of background probes and provides a probability that value of the signal intensity if part of the background noise [26]. Different normalization methods and cutoffs are used in miRNA expression analysis depending on the expression level of the miRNAs in the sample analysed [27,28]. After normalization and after applying a pvalue cutoff ≤0.05, differentially expressed miRNAs were evaluated after applying fold changes ≥2, 1.5 and 1.2. Due to the low number of miRNA displaying a fold change ≥2, fold-change of 1.5 and 1.2 were used as an arbitrary threshold to perform pathway enrichment analysis in order to identify the pathways that are overall affected by ALS ADEVs.

To elucidate pathways targeted by dysregulated miRNAs three online analytical tools were used, Database for Annotation, Visualization and Integrated Discovery (DAVID) [29], DIANA-mirPath [30] and miRSystem [31]. MiRSystem generates a list of enriched pathways that are regulated by queried miRNAs by incorporating miRNA expression values and matching miRNAs with the latest annotation [31]. It combines seven different algorithms and two validated databases to identify target genes and uses five pathway databases to characterize the enriched pathways. DIANA-miRPath performs miRNA pathway analysis and allows queried miRNAs and target genes to be visualized on pathway maps [30]. DAVID is a widely cited and used tool that discovers enriched functional-related gene groups and allows them to be visualized on pathway maps [29]. DAVID and DIANA-miRPath utilize enrichment *p*-values while miRSystem uses ranking scores based on the affinity of the interactions between miRNAs and target genes [31]. Five pathways that were common to more than two tools were found. From the five common pathways listed for up and downregulated miRNAs, two pathways that were of our interest were selected and genes that are involved in those pathways were found. The most enriched miRNAs were found by selecting genes, involved in the pathways of interest that were commonly found between the analytic tools.

### 2.10 Post mortem tissue

Autopsy donations to the Sheffield Brain Tissue Bank were performed with the written consent of the next of kin for the use of tissues for scientific research. Slices of brain and segments of spinal cord were frozen on copper plates in liquid nitrogen vapour and stored at −80 °C.

Six samples were retrieved from deep freezing (Table 3), and samples of motor cortex, spinal cord and lateral corticospinal tract taken by a qualified neuropathologist.

**Table 3:** Details of post-mortem tissue donors, including gender, age, post-mortem delay (PMD) and diagnosis.

Case ID	Gender	Age	PMD	Diagnosis
C1	F	63	31 h	Carcinoid Tumor
C2	M	63	25 h	Mesolthelioma
C3	M	67	63 h	Hepatocellular carcinoma
sALS1	M	66	40 h	sALS
sALS2	F	63	36 h	sALS
sALS3	M	66	19 h	sALS

The remaining nervous system tissue was formalin fixed for diagnostic confirmation and characterisation. The Sheffield Brain Tissue Bank Management Board gave ethical approval for use of tissue in this study under the provision to act as a Research Tissue Bank as approved by the Scotland Research Ethics Committee (ref. 08/MRE00/103).

### 2.11 Statistical analysis

All statistical tests were conducted using Graph Pad Prism 7 software (RRID:SCR_002798). Statistical analysis was performed by either Student's *t*-test, one-way ANOVA with Tukey post-hoc analysis, or two-way ANOVA with Sidak post-hoc analysis, depending on the number of variables in the respective experiment. All experiments were performed a minimum of three times. *p* < .05 was considered statistically significant. All *p* values and n values are documented in the figure legends.

## 3 Results

### 3.1 Conditioned medium and extracellular vesicles from C9orf72 astrocytes are toxic to MNs

We previously showed that C9orf72 iAstrocytes derived through direct conversion of fibroblasts into iNPCs induced a decrease in survival in Hb9-GFP^+^ mouse MNs in co-culture compared to non-ALS astrocytes [16]. Here we tested the effect of iAstrocyte conditioned medium on MN monocultures, to determine if secreted factors are main players in C9-ALS astrocytic-mediated neurotoxicity as previously reported for other genetic subtypes [15,17].

**Fig. 1:**
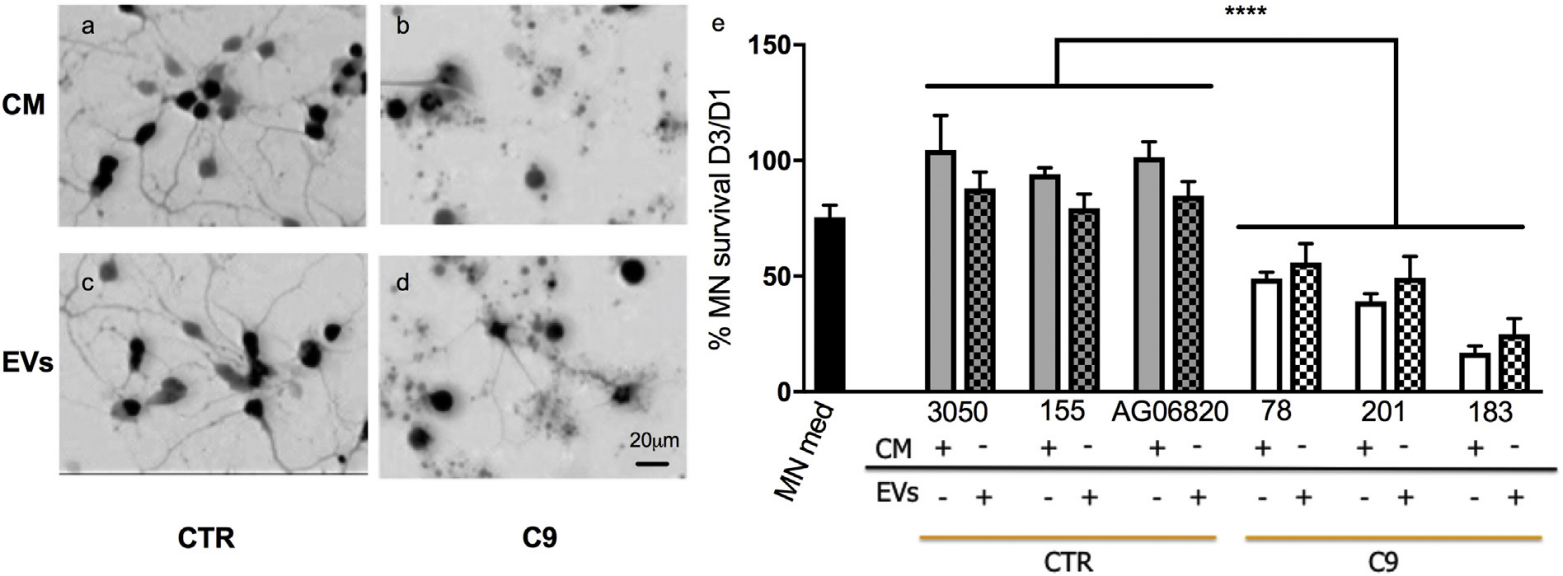
Conditioned medium (CM) and extracellular vesicles (EVs) from C9-ALS iAstrocytes induce MN death. Representative images of Hb9-GFP^+^MN monocultures treated for 72 h with astrocyte conditioned medium from control (CTR) or C9-ALS (C9) astrocytes (a and b respectively) or EVs isolated from the same conditioned media (c and d respectively). Quantification of HB9-GFP^+^MNs with axons performed after 3 days of treatment reveals that conditioned medium (solid bars) and EV treatment (checked bars) have similar effects on MN monocultures (e), with C9-ALS astrocyte medium and EVs inducing significantly lower MN survival. Survival of MN monocultures in MN medium (black solid bar) used as comparative reference (*n* = 3). Two-way ANOVA (*p* < .0001), 3xControls *versus* 3xC9-ALS, *N* = 3 per condition, error bar = SD. Scale bar = 20 μm.

Consistent with previous data, C9-ALS astrocyte conditioned medium treatment of MN monocultures resulted in 50–75% increased MN death compared to controls after 72 h (Fig. 1a, b, e; *p* < .0001 (Two-way ANOVA)). Since extracellular vesicles (EVs) have been implicated in cell-to-cell communication in a number of neurodegenerative diseases [32], we decided to determine their role in astrocyte-mediated MN death in C9-ALS. We isolated EVs from C9 iAstrocyte conditioned medium (10 ml) *via* ultracentrifugation, resuspended them in fresh non-conditioned astrocyte medium (10 ml) or complete MN medium with growth factors (10 ml) and we then treated the MN monocultures. Our data reveal that C9-EVs are equally toxic as the astrocyte conditioned medium (Fig. 1c-e, p < .0001 (Two-way ANOVA)). Dilution of the EVs in complete MN medium containing BDNF, GDNF and CNTF still resulted in MN death, proving that EVs are toxic to MNs (Supplementary Fig. 1). We also report here that ADEVs isolated from control astrocytes are consistently, even if not statistically significant, less supportive compared to whole conditioned medium (Fig. 1e) while C9-EVs are consistently less toxic than whole conditioned medium. This suggests that other factors not packaged into ADEVs contribute to MN health/death. Consistent with these findings, MN medium alone is not as supportive as astrocyte-conditioned medium from non-ALS controls (Fig. 1e and Supplementary Fig. 2a, b), proving that astrocytes have an active role in supporting neurite growth and synaptic formation.

### 3.2 EV biogenesis is impaired in C9orf72 astrocytes

**Fig. 2:**
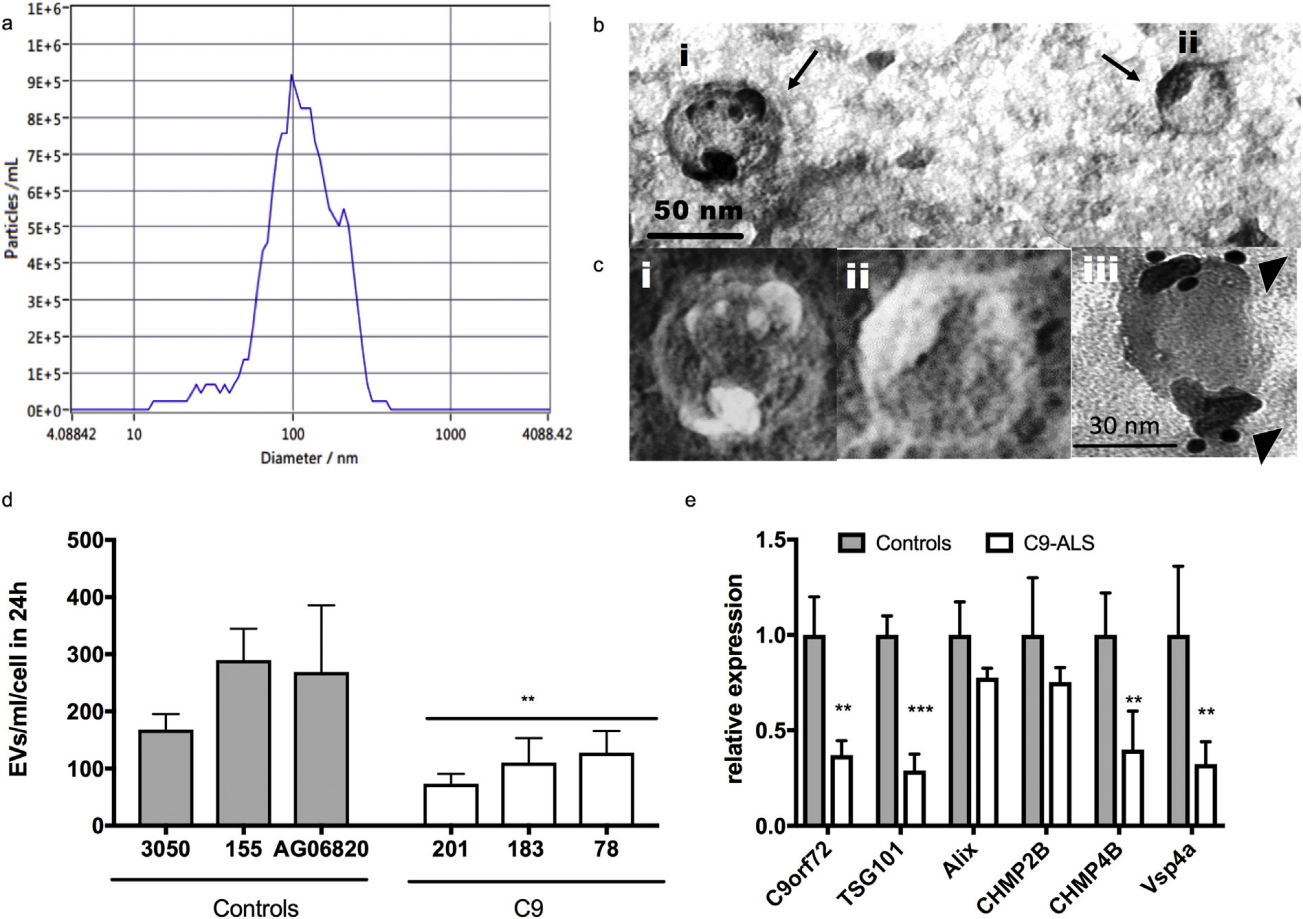
EV biogenesis is impaired in C9-ALS iAstrocytes. Representative size distribution graph (a) of ADEVs and TEM (b, c-i, c-ii) show that most ADEVs range between 50 and 120 nm. ADEVs are positive for CD63 (arrow heads) with immunogold labeling (c-iii). ADEV direct quantification using the ZetaView Nanoparticle Tracking System (d) shows a significant decrease in the number of particles secreted by C9 iAstrocytes (One-way ANOVA; *p* < .01). This suggests impairment in vesicle formation, as supported by TaqMan qRT-PCR data showing downregulation in a number of transcripts involved in EV biogenesis in C9 iAstrocytes (e). *t*-test (**p < .01; *** *p* < .001), 3xControls *versus* 3xC9-ALS, *N* = 3 per condition, error bar = SD.

Having assessed the detrimental effect of EVs secreted by C9-ALS astrocytes, we sought to characterize their abundance in the conditioned medium. We used the ZetaView Nanoparticle Tracking Analysis (NTA) system to identify particle size and number in conditioned medium from 3 controls and 3 C9-ALS patients. Quantification revealed no difference in the overall size range of EVs secreted by controls or patients, typically ranging between 70 and 200 nm. Most particles range between 50 and 120 nm, with a clear peak at 100 nm, indicating that exosomes are the main component of the total EV pool (Fig. 2a). We confirmed ADEV isolation by transmitted electron microscopy (TEM) and immunogold staining for CD63 (Fig. 2b and c).

**Fig. 3:**
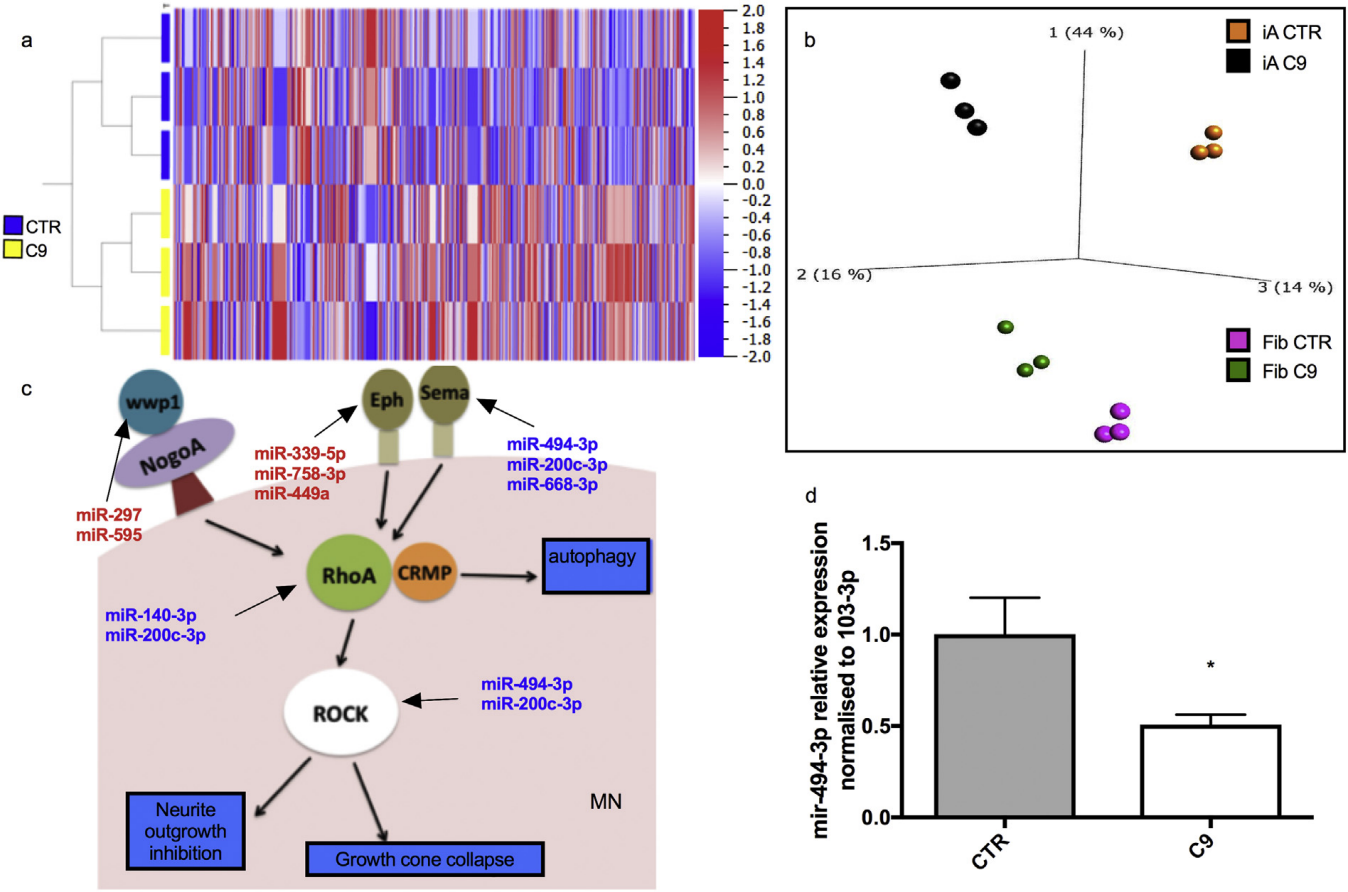
MicroRNAs regulating axonal maintenance are selectively dysregulated in C9 ADEVs. MiRNAs secreted by C9 and Control (CTR) iAstrocytes identify two separate groups in the hierarchical clustering analysis (a). The PCA plot (b) shows how EV-secreted miRNAs differentiate iAstrocytes (iA) and fibroblasts (Fib) on one axis and controls (CTR) and C9 patients (C9) on another axis. iA also show more dramatic differences between patients and controls compared to fibroblasts. Pathway analysis of the dysregulated miRNAs identifies axonal growth and maintenance as the most affected pathway (c). Downregulated (in blue) miRNAs target Semaphorins, RhoA and Rock, thus predicting an increase in these proteins, which would lead to growth cone collapse. Upregulated miRNAs (in red) target Ephs and Wwp1, which would lead to their downregulation. Wwp1 inactivates NogoA, thus this would also lead to axonal collapse. TaqMan qRT-PCR confirms significant downregulation of miR-494-3p, which negatively regulates Sema3A (d). *N* = 3 per group; error bar = SD. Two-tail unpaired *t*-test (*p* < .05). (For interpretation of the references to colour in this figure legend, the reader is referred to the web version of this article.)

Interestingly, ADEV quantification showed that iAstrocytes from C9 patients secrete fewer vesicles than healthy individuals across a range of vesicle sizes (Fig. 2d and Fig. 3). It was recently reported that C9orf72 not only is involved in autophagy [13], but also in vesicle trafficking [11]. For this reason, we decided to assess the mRNA expression level of proteins involved in EV formation and processing. Consistent with the observed reduction in EV secretion, qRTPCR analysis showed that transcripts encoding for *C9orf72, TSG101, CHMP4B and VSP4A* are significantly downregulated in C9-ALS iAstrocytes (Fig. 2e).

### 3.3 MicroRNAs regulating axonal maintenance are selectively dysregulated in C9 patient ADEVs

Recent studies have provided strong evidence that astrocytes can regulate neuronal health through EV-secreted miRNAs and indeed we found that between 30 and 80% of the iAstrocyte EV RNA cargo is composed of miRNAs (Supplementary Table 1). Therefore, we proceeded to interrogate the panel of miRNAs secreted *via* ADEVs by 3 control and 3 C9-ALS iAstrocyte lines using *Affymetrix* miRNA Microarray Chips.

The miRNA expression levels were determined using Expression Console (Affymetrix) and the number of miRNAs detected above background (DABG) was 6632. Hierarchical clustering and principal component analysis (PCA) of the detected miRNAs clearly identified patients and controls as two separate groups (Fig. 3a, b). We interrogated the data for differentially expressed transcripts by setting a threshold with *p*-value ≤.05 and fold change ≥1.5 we identified 64 dysregulated miRNAs (51 upregulated and 13 downregulated). We then applied a less stringent fold-change ≥1.2 to ensure consistency within our results, as one would expect that the pathways identified with more stringent criteria would still be present when the criteria are relaxed to allow performing a broader pathway enrichment analysis. This less stringent analysis identified 193 differentially expressed miRNAs, 116 were upregulated whilst 77 were downregulated (Supplementary Table 2).

In order to identify transcripts that are targeted by dysregulated miRNAs, two different web-based analytic tools, DIANAmiRPath and miRSystem were used, as they are based on different algorithms and databases [30,31]. To interrogate the enriched pathways targeted by the dysregulated miRNAs, we then used miRSystem and DAVID, which use different ranking systems. Pathway identification and ranking through different algorithms gave us confidence that common hits would be statistically robust. Of the top 5 pathways identified by these two tools, axonal guidance and maintenance as well as adherens junctions were identified through both approaches and regardless of the fold change cutoff applied (Supplementary Table 3).

To identify which pathways might be dysregulated in multiple cell types affected by the C9 mutation, *versus* pathways that are astrocyte-specific, hence potentially involved in MN death, we interrogated the miRNA profile of EVs secreted by C9-ALS fibroblasts compared to unaffected controls through the same workflow. The PCA identified 4 groups, clearly separating the miRNA profile of fibroblasts from iAstrocytes and control and patients within each group (Fig. 3b). We found that, while dysregulation of adherens junctions is a category present in both data sets, axonal guidance and maintenance is unique to the iAstrocytes with 13 dysregulated miRNAs involved in the pathway (Fig. 3c). Through pathway analysis, we identified hsa-miR-494-3p as an upstream target in regulating axonal maintenance, with its primary target being Semaphorin 3A (Sema3A). Increased Sema3A expression has been recently described in *post mortem* motor cortex of sporadic ALS patients [33]. MiR-494-3p was also the most dysregulated miRNA identified in the axonal maintenance pathway (microarray data: fc = −2.38, *p* = .047 (One-way ANOVA)) and its downregulation was validated *via* TaqMan qPCR (Fig. 3d).

### 3.4 MiR-494-3p mimic restores SEMA3A levels and increases MN survival

**Fig. 4:**
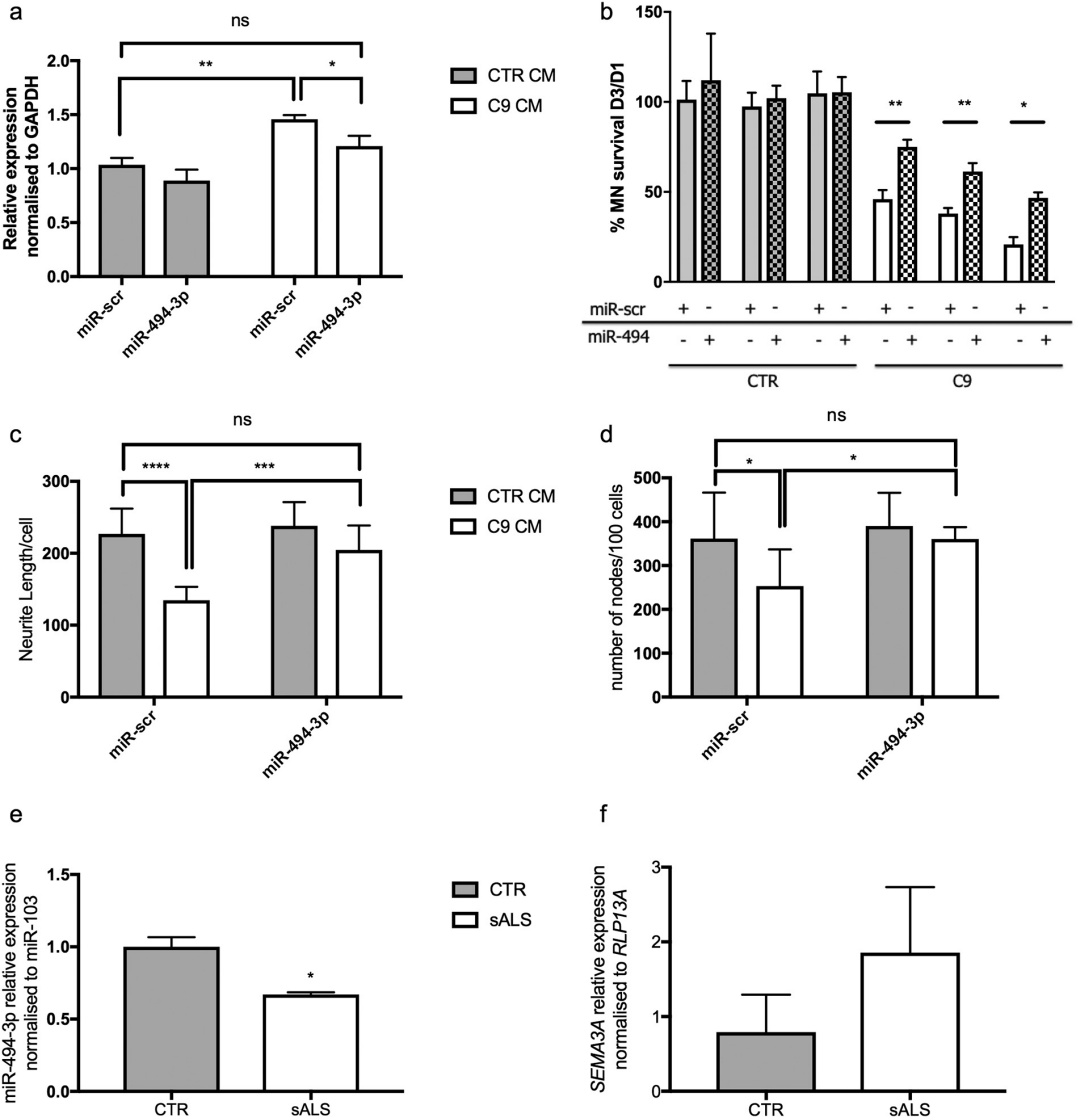
MiR-494-3p mimic restores *SEMA3A* levels and increases MN survival. QRT-PCR results show that HB9-GFP^+^ MNs treated with C9 iAstrocyte conditioned medium supplemented with a scramble miRNA (miR-scr) display elevated levels of *Sema3A* compared to control iAstrocyte conditioned medium treatment + miR-scr (a). *Sema3A* levels, however, are downregulated after 48 h treatment with a miR-494-3p mimic (a). *N* = 3 per condition; Error bars = SD. Decrease in *Sema3A* corresponds with improvement in MN survival (b), increase in neurite length (c) and number of nodes/intersection in MN monocultures (d) treated with C9 iAstrocyte conditioned medium plus miR-494-3p. MN measurements were performed 72 h post-treatment. In b N = 3 per condition; in c and d 3 *N* = 9 (independent replicates for each of the 3 patients and 3 controls); error bar = SD. Statistical analysis: Two-way ANOVA with multiple comparison test and Sidak correction (*p < .05; **p < .01; ***p < .001; ****p < .0001). QRT-PCR analysis of lateral cortico-spinal tract tissue from 3 non-ALS and 3 sporadic ALS patients with limb onset showed a significant 30% reduction in miR-494-3p (e) (two-tailed *t*-test, p = .02), while no significant change was detected in *SEMA3A* (f) (two-tailed t-test *p* = .08).

In order to test the effect of miR-494-3p on its identified target, *i.e.* motor neuronal *Sema3A*, we treated HB9-GFP^+^ mouse MN monocultures with conditioned medium from control or C9-ALS iAstrocytes supplemented with either a scramble miRNA (miR-scr) or a miR-494-3p mimic and then we evaluated the expression level of *Sema3A via* qRT-PCR. Mouse HB9-GFP^+^ MNs could be used because miR-494-3p is highly conserved between mouse and human. The results show that treatment with C9 conditioned medium indeed significantly increased the levels of *Sema3A* mRNA in HB9-GFP^+^ MN by 46% after only 48 h of treatment (Fig. 4a). Indeed, treatment of the MNs with a miR-494-3p mimic in the presence of C9 iAstrocyte conditioned medium significantly reduced the levels of *Sema3A* by 25% in the MNs (Fig. 4a).

We then assessed the functional effect of this transcriptional regulation to verify the link between miR-494-3p downregulation and astrocyte-mediated MN death. We cultured HB9-GFP^+^ mouse MNs and we treated them with conditioned medium from controls or C9 patients supplemented with either a miR-scr or miR-494-3p mimic. We then measured MN number (Fig. 4b), neurite length (Fig. 4c) and number of nodes/intersections (Fig. 4d) to assess not only MN survival over time, but also branching and neurite tree complexity, which is expected to be directly affected by Sema3A expression.

As expected MNs treated with C9 conditioned medium displayed significantly lower MN survival, neurite length and number of nodes per cell (Fig. 4b-d) compared to controls, however, miR-494-3p treatment led to a complete rescue in neurite length and number of nodes per cell (Fig. 4c, d), accompanied by a significant 20–25% increase in MN survival compared to miR-scr treated cells (Fig. 4b). Comparable results were obtained when MN monocultures were treated with ADEVs isolated from the iAstrocyte-conditioned medium and then resuspended in fresh non-conditioned medium supplemented with either miR-scr or miR-494-3p (data not shown).

In order to evaluate the relevance of our *in vitro* data in human disease, we assessed the expression levels of miR-494-3p and *SEMA3A* in the motor cortex and lateral cortico-spinal tract of the spinal cord in post-mortem tissues from sALS patients. We detected significantly lower levels of miR-494-3p (*p* = .02, Two-tailed *t*-test), while SEMA3A did not show a significant increase as predicted in the cortico-spinal tract when comparing 3 sporadic ALS (sALS) patients to 3 non-ALS controls (Fig. 4e, f).

## 4 Discussion

Neurodegenerative disorders are extremely complex diseases characterized by high heterogeneity and interplay between different cell types. Discoveries of the past 20 years have highlighted that ALS is part of a disease spectrum ranging from frontotemporal dementia (FTD) to pure motor neurone disease, it is multifactorial in origin, with contribution of genetic, epigenetic and environmental factors and it unravels through a yet undeciphered communication between neuronal and non-neuronal cells [34,35]. Astrocytes have multiple functions, from maintaining tissue homeostasis to supporting neurite growth and remodeling during development as well as maintenance during adulthood (reviewed by Clarke and Barres, 2013) [36].

Although massive progress has been made in understanding the chemical signals that regulate the motor neuron-astrocyte crosstalk, very little is known about the role of micro RNAs (miRNAs) in this communication.

Work carried out by us and others has shown that astrocytes are major players in ALS pathology, (reviewed by Ferraiuolo, 2014) [37]. In particular, it has been shown that astrocytes derived from either post-mortem tissues or fibroblasts from ALS patients are toxic to MNs and this toxicity is also transferred through conditioned medium [16,17].

ADEV cargo has been interrogated before as a culprit not only for motor neurone injury, but also disease spreading, as mutant SOD1 was detected in the exosomes secreted by primary astrocytes overexpressing the mutant SOD1 gene [38].

Several recent studies, however, have turned their attention towards EV miRNA, rather than protein, cargo. Most of these studies have been performed in rodent models overexpressing mutant SOD1 and have identified motor neuron-secreted miRNAs regulating astrocytic function [24].

On the contrary, in the present study, we focused on astrocytes and on understanding how secreted ADEVs might contribute to MN degeneration. We report that induced astrocytes (iAstrocytes) derived from human fibroblasts secrete less EVs than unaffected controls, consistent with recent data collected in C9orf72 knockdown cell models and iPSC-derived neurons from patients [11]. This characteristic seems to be specific to the role of C9orf72, as primary mouse astrocytes expressing mutant SOD1 were reported to secrete more exosomes than controls [38].

Of great relevance for the EVs field, we also observed that EV secretion decreases with cell density *in vitro*, thus highlighting the importance of monitoring cell growth parameters and keeping them consistent across conditions.

Our data show that ADEVs isolated from iAstrocyte conditioned medium are sufficient to cause MN death even in presence of trophic factors, thus demonstrating that ADEVs carry toxic factors.

Moreover, our miRNA profiling shows ADEVs from ALS patients and unaffected controls carry a unique miRNA cargo distinct from the fibroblasts of origin, which are, in fact, not toxic to MNs. Interestingly for astrocytes that have not been primed with motor neurons, our data show that iAstrocytes secrete miRNAs regulating a number of transcripts encoding proteins involved in axonal growth and maintenance, similarly to primary astrocytes [23]. This indicates that this regulatory program of miRNA expression is intrinsic to this cell type and altered in C9ORF72-ALS.

Consistent with this observation, HB9-GFP^+^ wild-type mouse motor neurons treated with motor neuron medium display shorter neurites and less complex projection network than motor neurons treated with conditioned medium from unaffected controls (Supplementary Fig. 2). Additionally, conditioned medium from C9-ALS astrocytes causes axonal shortening before motor neuron cell body loss.

Our study has identified several miRNAs involved in regulating axonal/neurite maintenance and growth, in particular, we have identified significant downregulation of miR-494-3p, a miRNA involved in the regulation of several genes, including SEMA3A. Our data demonstrate that astrocyte-secreted miR-494-3p manipulation is effective in regulating *SEMA3A* neuronal levels *in vitro* and that its upregulation through an engineered miRNA mimic rescues both neurite length and network complexity, as well as motor neuron survival.

Recent studies have also shown that Sema3A increase in ALS is not limited to the periphery [39], but it is also a central phenomenon affecting motor neurons in the motor cortex of sporadic ALS patients [33], astrocytes in cases of spinal cord injury [40] and oligodendrocytes in multiple sclerosis [41].

Elegant studies, however, have highlighted the complex role of SEMA3A in axonal development, positioning and maintenance [20] in CNS and have reported that under normal conditions specifically astrocyte-secreted SEMA3A has a pro-survival role on spinal MN rather than detrimental, while the opposite is true for cortical neurons [42]. In our study, however, we have not investigated the levels of SEMA3A secreted by astrocytes and its role on MN survival.

**Fig. 5:**
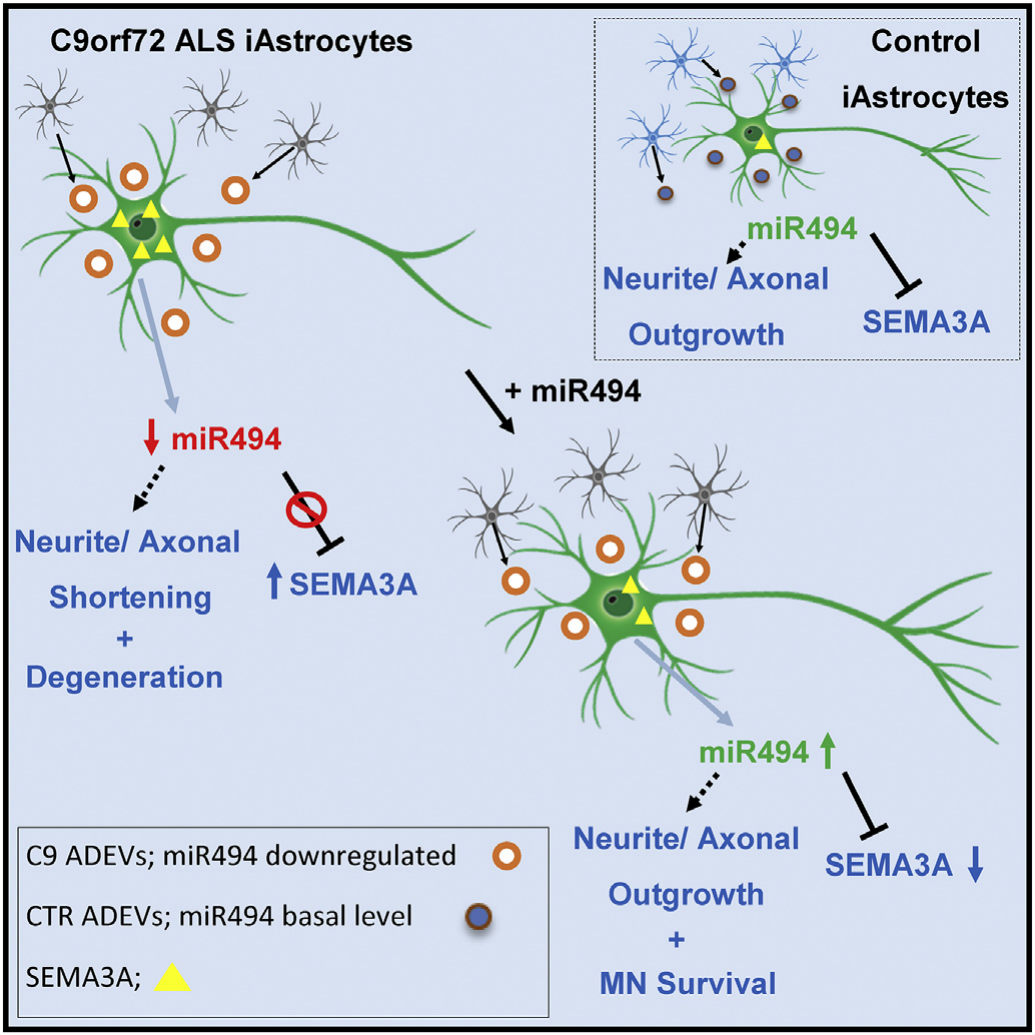
Graphic summary of the study. Our study shows that iAstrocytes from C9orf72 patients and controls secrete ADEVs containing miRNAs that affect MN survival and neurite growth. In particular ADEVs from C9orf72 iAstrocytes cause neurite and axonal shortening and MN death. This toxic effect is partly caused by lower levels of miR-494-3p secreted by C9orf72 iAstrocytes. A miRNA mimic of this molecule can restore MN survival and axonal maintenance. In addition, miR-494-3p has the ability to inhibit Sema3A expression in MN, which is, in fact, extremely low in healthy neurons.

Our *in vitro* data indicate that intrinsic high levels of SEMA3A in MNs are associated with neurite retraction and MN death and exogenous miR-494-3p is able to decrease *Sema3A* levels. The MN rescue observed in cultures treated with C9-ALS ADEV supplemented with miR-494-3p, however, is likely to be the result of the action of miR-494-3p on several targets acting upon axonal maintenance and MN survival rather than solely regulation on motor neuronal semaphorin (Fig. 5).

Neuronal degeneration and synaptic loss are phenomena affecting both the CNS as well as the NMJ and they can be identified in clinic at diagnosis. Because not all the synapses are affected or lost at the same time, their preservation is an appealing intervention even after symptom onset.

In our *in vitro* system, unfortunately, we were not able to test the effect of miR-494-3p selectively on the axon as opposed to the neurite tree, which would have clarified whether the crosstalk happening centrally in the motor cortex and spinal cord has an effect also on the periphery, *i.e.* the NMJ. It is likely that the stimuli applied by the astrocytes in the brain and spinal cord on the MN cell bodies have a deep impact on the axons and the distal neuronal cell compartment. Future *in vitro* experiments should utilize microfluidic chambers, where the two cell compartments can be kept separated and the effect of ADEVs can be tested onto neurites or axons separately.

Using post-mortem tissue from sALS patients, we show here that within the cortico-spinal tract, the motor pathway formed by the descending axons of the primary cortical MNs in the motor cortex towards the lower MNs in the brainstem and spinal cord, we have detected a decrease in miR-494-3p. These data support the potential role of this miRNA not only in the context of C9-ALS pathology, but the wider sALS population.

The lack of specificity that characterizes miRNAs might pose a limitation in terms of safety concerns for *in vivo* manipulation, however, engineered miRNAs might satisfy the need to target multiple molecules in the same or different pathways at the same time. Gene therapy approaches targeting single genes have been proven safe and efficacious in the field of neuromuscular disorders [44,45], supporting the idea that targeted and timed gene manipulation could be developed as a potential therapeutic approach.

This study is of great relevance not only for the field of ALS, but potentially other neurodegenerative conditions, where miR-494-3p expression might be used to preserve neurite/axonal health.

## Authors contribution

A.V. and M.A.M. conducted the *in vitro* experiments; L.M.C. contributed to the miRNA mimics experiments; B.O., Y.K., J.T. and I.N. conducted the microarray experiments and data analysis, S.N. conducted the TEM experiments, M.J.S. and L.F. contributed to data analysis, L.F. designed the experiments with contributions from P.R.H. and G.M.H., L.F. wrote the paper with feedback from all authors.

## Acknowledgments/funding

We would like to thank Dr. Stuart Hunt and the ZetaView facility for their help with EV characterisation, the EM facility at the University of Sheffield for their training and support, the Sheffield Brain bank and Dr. Robin Highley for their support with the human post-mortem tissue.

A.V., G.M.H. and L.F. are funded by the Thierry Latran Fondation FTLAAP2016/ FERRAIUOLO/ Astrocyte secretome. S.N., L.F, L.M.C. and G.M.H. are funded by the MND Association grants Bonanno/Apr16/848-791 and Hautbergue/Apr16/846-79. G.M.H. is also funded by the MRC NIRG MR/R024162/1 and Royal Society International Exchanges grant IEC\R3\170,103. L.F. is also funded by the AMS/Wellcome Trust. The funders had no role in study design, data collection, data analysis, interpretation and writing of the report.

## Conflict of interest

Dr. Ferraiuolo reports grants from Thierry Latran Foundation, grants from Academy of Medical Sciences, during the conduct of the study. The authors declare no other relationships/conditions/circumstances that present a potential conflict of interest.

## Appendix A Supplementary data

Supplementary material Image 1

## References

[1] Fischer L.R., Culver D.G., Tennant P. (2004). Amyotrophic lateral sclerosis is a distal axonopathy: evidence in mice and man. Exp Neurol.

[2] Walsh M.J., Cooper-Knock J., Dodd J.E. (2015). Invited Review: Decoding the pathophysiological mechanisms that underlie RNA dysregulation in neurodegenerative disorders: a review of the current state of the art. Neuropathol Appl Neurobiol.

[3] Renton A.E., Chiò A., Traynor B.J. (2014). State of play in amyotrophic lateral sclerosis genetics. Nat Neurosci.

[4] Emde A., Eitan C., Liou L.-L. (2015). Dysregulated miRNA biogenesis downstream of cellular stress and ALS-causing mutations: a new mechanism for ALS. EMBO J.

[5] Haramati S., Chapnik E., Sztainberg Y. (2010). miRNA malfunction causes spinal motor neuron disease. Proc Natl Acad Sci U S A.

[6] Hawley Z.C.E., Campos-Melo D., Droppelmann C.A., Strong M.J. (2017). MotomiRs: miRNAs in motor neuron function and disease. Front Mol Neurosci.

[7] DeJesus-Hernandez M., Mackenzie I.R., Boeve B.F. (2011). Expanded GGGGCC hexanucleotide repeat in noncoding region of C9ORF72 causes chromosome 9p-linked FTD and ALS. Neuron.

[8] Renton A.E., Majounie E., Waite A. (2011). A hexanucleotide repeat expansion in C9ORF72 is the cause of chromosome 9p21-linked ALS-FTD. Neuron.

[9] Hautbergue G.M., Castelli L.M., Ferraiuolo L. (2017). SRSF1-dependent nuclear export inhibition of C9ORF72 repeat transcripts prevents neurodegeneration and associated motor deficits. Nat Commun.

[10] Prudencio M., Belzil V.V., Batra R. (2015). Distinct brain transcriptome profiles in C9orf72-associated and sporadic ALS. Nat Neurosci.

[11] Aoki Y., Manzano R., Lee Y. (2017). C9orf72 and RAB7L1 regulate vesicle trafficking in amyotrophic lateral sclerosis and frontotemporal dementia. Brain.

[12] Boeynaems S., Bogaert E., Kovacs D. (2017). Phase separation of C9orf72 dipeptide repeats perturbs stress granule dynamics. Mol Cell.

[13] Webster C.P., Smith E.F., Bauer C.S. (2016). The C9orf72 protein interacts with Rab1a and the ULK1 complex to regulate initiation of autophagy. EMBO J.

[14] Yamanaka K., Chun S.J., Boillee S. (2008). Astrocytes as determinants of disease progression in inherited amyotrophic lateral sclerosis. Nat Neurosci.

[15] Haidet-Phillips A.M., Hester M.E., Miranda C.J. (2011). Astrocytes from familial and sporadic ALS patients are toxic to motor neurons. Nat Biotechnol.

[16] Meyer K., Ferraiuolo L., Miranda C.J. (2014). Direct conversion of patient fibroblasts demonstrates non-cell autonomous toxicity of astrocytes to motor neurons in familial and sporadic ALS. Proc Natl Acad Sci U S A.

[17] Re D.B., Le Verche V., Yu C. (2014). Necroptosis drives motor neuron death in models of both sporadic and familial ALS. Neuron.

[18] Ferraiuolo L., Higginbottom A., Heath P.R. (2011). Dysregulation of astrocyte–motoneuron cross-talk in mutant superoxide dismutase 1-related amyotrophic lateral sclerosis. Brain.

[19] Kia A., McAvoy K., Krishnamurthy K., Trotti D., Pasinelli P. (2018). Astrocytes expressing ALS-linked mutant FUS induce motor neuron death through release of tumor necrosis factor-alpha. Glia.

[20] Molofsky A.V., Kelley K.W., Tsai H.-H. (2014). Astrocyte-encoded positional cues maintain sensorimotor circuit integrity. Nature.

[21] Gosselin R.-D., Meylan P., Decosterd I. (2013). Extracellular microvesicles from astrocytes contain functional glutamate transporters: regulation by protein kinase C and cell activation. Front Cell Neurosci.

[22] Schratt G. (2009). microRNAs at the synapse. Nat Rev Neurosci.

[23] Chaudhuri A.D., Dastgheyb R.M., Yoo S.-W. (2018). TNFα and IL-1β modify the miRNA cargo of astrocyte shed extracellular vesicles to regulate neurotrophic signaling in neurons. Cell Death Dis.

[24] Hoye M.L., Regan M.R., Jensen L.A. (2018). Motor neuron-derived microRNAs cause astrocyte dysfunction in amyotrophic lateral sclerosis. Brain.

[25] Maimon R., Ionescu A., Bonnie A. (2018). miR126-5p down-regulation facilitates axon degeneration and NMJ disruption via a non-cell-autonomous mechanism in ALS. J Neurosci.

[26] Clark T.A., Schweitzer A.C., Chen T.X. (2007). Discovery of tissue-specific exons using comprehensive human exon microarrays. Genome Biol.

[27] Pradervand S., Weber J., Thomas J. (2009). Impact of normalization on miRNA microarray expression profiling. RNA.

[28] Wang B., Xi Y. (2013). Challenges for MicroRNA microarray data analysisf. Microarrays.

[29] Huang D.W., Sherman B.T., Lempicki R.A. (2009). Systematic and integrative analysis of large gene lists using DAVID bioinformatics resources. Nat Protoc.

[30] Vlachos I.S., Zagganas K., Paraskevopoulou M.D. (2015). DIANA-miRPath v3.0: deciphering microRNA function with experimental support. Nucleic Acids Res.

[31] Lu T.-P., Lee C.-Y., Tsai M.-H. (2012). miRSystem: an integrated system for characterizing enriched functions and pathways of MicroRNA targets. PLoS One..

[32] Thompson A.G., Gray E., Heman-Ackah S.M. (2016). Extracellular vesicles in neurodegenerative disease — pathogenesis to biomarkers. Nat Rev Neurol.

[33] Körner S., Böselt S., Wichmann K. (2016). The axon guidance protein semaphorin 3A is increased in the motor cortex of patients with amyotrophic lateral sclerosis. J Neuropathol Exp Neurol.

[34] Ferraiuolo L., Meyer K., Sherwood T.W. (2016). Oligodendrocytes contribute to motor neuron death in ALS via SOD1-dependent mechanism. Proc Natl Acad Sci U S A.

[35] Hardiman O., Al-Chalabi A., Chio A. (2017). Amyotrophic lateral sclerosis. Nat Rev Dis Prim.

[36] Clarke L.E., Barres B.A. (2013). Erratum: Emerging roles of astrocytes in neural circuit development. Nat Rev Neurosci.

[37] Ferraiuolo L. (2014). The non-cell-autonomous component of ALS: new in vitro models and future challenges. Biochem Soc Trans.

[38] Basso M., Pozzi S., Tortarolo M. (2013). Mutant copper-zinc superoxide dismutase (SOD1) induces protein secretion pathway alterations and exosome release in astrocytes: implications for disease spreading and motor neuron pathology in amyotrophic lateral sclerosis. J Biol Chem.

[39] De Winter F., Vo T., Stam F.J. (2006). The expression of the chemorepellent Semaphorin 3A is selectively induced in terminal Schwann cells of a subset of neuromuscular synapses that display limited anatomical plasticity and enhanced vulnerability in motor neuron disease. Mol Cell Neurosci.

[40] Kaneko S., Iwanami A., Nakamura M. (2006). A selective Sema3A inhibitor enhances regenerative responses and functional recovery of the injured spinal cord. Nat Med.

[41] Gutiérrez-Franco A., Costa C., Eixarch H. (2016). Differential expression of sema3A and sema7A in a murine model of multiple sclerosis: Implications for a therapeutic design. Clin Immunol.

[42] Birger A., Ottolenghi M., Perez L., Reubinoff B., Behar O. (2018). ALS-related human cortical and motor neurons survival is differentially affected by Sema3A article. Cell Death Dis.

[44] Iannitti T., Scarrott J.M., Likhite S. (2018). Translating SOD1 Gene Silencing toward the Clinic: a Highly Efficacious, Off-Target-free, and Biomarker-Supported Strategy for fALS. Mol Ther - Nucleic Acids.

[45] Mendell J.R., Al-Zaidy S., Shell R. (2017). Single-dose gene-replacement therapy for spinal muscular atrophy. N Engl J Med.

