# Supplementary material for: Micro-RNAs secreted through astrocyte-derived extracellular vesicles cause neuronal network degeneration in C9orf72 ALS
Source: eBioMedicine. 2019 Jan 31;40:626–35. doi: 10.1016/j.ebiom.2018.11.067 (PMC6413467; doi:10.1016/j.ebiom.2018.11.067)
Supplement: Supplementary file 1 — Supplementary material [file mmc1.docx]

**Supplementary Figures and Tables**

**Supplementary Fig. 1** ADEVs from C9-ALS iAstrocytes diluted in MN medium supplemented with growth factors are toxic to Hb9-GFP^+^ MN, causing a decrease in MN survival. This indicates that C9-ALS ADEVs carry toxic molecules rather than lacking supportive factors. N=3/condition and error bar=SD. One-way ANOVA

**Supplementary Fig. 2** Conditioned medium from healthy astrocytes actively supports neurite and axonal elongation as well as neuronal network (node) formation.

Neurite (a) and number of nodes (b) developed after 24h from plating in MN monocultures treated with conditioned medium from Control (CTR, gold bar) or C9-ALS iAstrocytes (green bar) or MN medium (blue bar) supplemented with growth factors. N=3/condition and error bar=SD. One-way ANOVA

**Supplementary Fig. 3** EV biogenesis is impaired in C9-ALS iAstrocytes. Graph representing the number of particles of various sizes (from 10 to 250nm)/ml secreted by 3 controls and 3 C9-ALS iAstrocytes. EVs of sizes from 50-150nm are less represented in C9-ALS compared to controls. N=3 per point (average shown)

**Supplementary Table 1.** ADEV are enriched in miRNAs. Related to Figure 3.

| **Cell line** | **Genotype** | **Small RNA Chip** |
| --- | --- | --- |
|  |  | **% miRNA**±**SD** |
| CTR AG EVs | Control | 87±2 |
| CTR AG iAstro | Control | 34±6 |
| CTR 155 EVs | Control | 88±2 |
| CTR 155 iAstro | Control | 33±5 |
| CTR 3050 EVs | Control | 89±3 |
| CTR 3050 iAstro | Control | 29±2 |
| C9_201 EVs | C9orf72 | 68±7 |
| C9_201 iAstro | C9orf72 | 35±5 |
| C9_183 EVs | C9orf72 | 75±5 |
| C9_183 iAstro | C9orf72 | 32±1 |
| C9_201 EVs | Control | 73±2 |
| C9_201 iAstro | Control | 23±4 |

**Supplementary Table 2.** List of differentially expressed miRNAs (p<0.05, fc>1.2) isolated from ADEVs. Related to Figure 3.

| **Transcript Cluster ID** | **Transcript ID** | **Fold Change (linear) (Patient vs. Control)** | **ANOVA p-value (Patient vs. Control)** |
| --- | --- | --- | --- |
| 20533699 | ENSG00000239063 | 11.1 | 0.039768 |
| 20533700 | ENSG00000239063 | 10.06 | 0.035177 |
| 20518818 | hsa-miR-4443 | 8.75 | 0.041028 |
| 20533283 | ENSG00000238433 | 8.63 | 0.027849 |
| 20518426 | hsa-miR-3907 | 7.26 | 0.005057 |
| 20504297 | hsa-miR-574-5p | 4.17 | 0.004705 |
| 20500799 | hsa-miR-195-3p | 3.9 | 0.011866 |
| 20536805 | hsa-mir-4525 | 3.84 | 0.005432 |
| 20525468 | hsa-miR-6753-3p | 3.8 | 0.038535 |
| 20519518 | hsa-miR-4701-3p | 3.61 | 0.007437 |
| 20518843 | hsa-miR-3135b | 3.5 | 0.012578 |
| 20535699 | hsa-mir-297 | 3.32 | 0.046221 |
| 20518898 | hsa-miR-4502 | 3.2 | 0.032108 |
| 20504432 | hsa-miR-660-3p | 3.09 | 0.03801 |
| 20506715 | hsa-miR-1183 | 3.06 | 0.040033 |
| 20515562 | hsa-miR-3148 | 2.88 | 0.014332 |
| 20537508 | hsa-mir-6765 | 2.86 | 0.009048 |
| 20537480 | hsa-mir-6736 | 2.79 | 0.00159 |
| 20519585 | hsa-miR-3064-5p | 2.75 | 0.029356 |
| 20519663 | hsa-miR-4436b-5p | 2.67 | 0.020494 |
| 20525623 | hsa-miR-6831-5p | 2.61 | 0.001294 |
| 20525673 | hsa-miR-6856-5p | 2.59 | 0.022404 |
| 20518835 | hsa-miR-4455 | 2.57 | 0.002857 |
| 20519642 | hsa-miR-4767 | 2.54 | 0.007228 |
| 20533490 | ENSG00000238745 | 2.49 | 0.007985 |
| 20525712 | hsa-miR-6875-3p | 2.32 | 0.019338 |
| 20525553 | hsa-miR-6796-5p | 2.19 | 0.016045 |
| 20533680 | ENSG00000239035 | 2.05 | 0.008713 |
| 20509226 | hsa-miR-1909-5p | 1.97 | 0.026263 |
| 20504332 | hsa-miR-595 | 1.95 | 0.020697 |
| 20536747 | hsa-mir-3689f | 1.85 | 0.004726 |
| 20520325 | hsa-miR-5088-5p | 1.79 | 0.031814 |
| 20536744 | hsa-mir-3689d-1 | 1.73 | 0.001056 |
| 20536745 | hsa-mir-3689d-2 | 1.73 | 0.001056 |
| 20537622 | hsa-mir-6876 | 1.73 | 0.003224 |
| 20500766 | hsa-miR-125a-3p | 1.71 | 0.003522 |
| 20535810 | hsa-mir-1204 | 1.7 | 0.018125 |
| 20534610 | hsa-mir-9-1 | 1.67 | 0.009895 |
| 20533492 | ENSG00000238746 | 1.62 | 0.040266 |
| 20533664 | ENSG00000239013 | 1.62 | 0.035526 |
| 20533507 | ENSG00000238767 | 1.6 | 0.019035 |
| 20534605 | hsa-mir-153-1 | 1.59 | 0.007808 |
| 20534885 | hsa-mir-339 | 1.59 | 0.035086 |
| 20519074 | hsa-miR-3972 | 1.58 | 0.009405 |
| 20504424 | hsa-miR-656-5p | 1.57 | 0.041355 |
| 20536654 | hsa-mir-550b-1 | 1.55 | 0.034001 |
| 20536655 | hsa-mir-550b-2 | 1.55 | 0.034001 |
| 20538145 | U19-2 | 1.54 | 0.005434 |
| 20537520 | hsa-mir-6777 | 1.52 | 0.00837 |
| 20537600 | hsa-mir-6855 | 1.52 | 0.013436 |
| 20532810 | ENSG00000200677 | 1.5 | 0.00823 |
| 20536186 | hsa-mir-3116-1 | 1.49 | 0.047946 |
| 20532681 | ACA47 | 1.47 | 0.026781 |
| 20525620 | hsa-miR-6829-3p | 1.47 | 0.037411 |
| 20537639 | hsa-mir-6893 | 1.47 | 0.014169 |
| 20533252 | ENSG00000238372 | 1.45 | 0.032921 |
| 20533508 | ENSG00000238767 | 1.45 | 0.031936 |
| 20519490 | hsa-miR-4685-3p | 1.43 | 0.047771 |
| 20535857 | hsa-mir-548f-5 | 1.43 | 0.020684 |
| 20515514 | hsa-miR-3120-5p | 1.42 | 0.007311 |
| 20534893 | hsa-mir-345 | 1.42 | 0.030283 |
| 20525590 | hsa-miR-6814-3p | 1.42 | 0.005202 |
| 20525744 | hsa-miR-6891-3p | 1.42 | 0.041557 |
| 20532713 | ACA67B | 1.41 | 0.013821 |
| 20533491 | ENSG00000238746 | 1.41 | 0.007853 |
| 20535757 | hsa-mir-1179 | 1.41 | 0.016519 |
| 20535961 | hsa-mir-1910 | 1.41 | 0.002583 |
| 20536645 | hsa-mir-3939 | 1.41 | 0.011455 |
| 20532811 | ENSG00000200677 | 1.4 | 0.038352 |
| 20532591 | 14qII-20 | 1.39 | 0.012512 |
| 20537510 | hsa-mir-6767 | 1.39 | 0.014716 |
| 20535795 | hsa-mir-1226 | 1.37 | 0.034706 |
| 20535098 | hsa-mir-449a | 1.37 | 0.011783 |
| 20535171 | hsa-mir-523 | 1.37 | 0.022353 |
| 20533773 | ENSG00000239188 | 1.36 | 0.019607 |
| 20536557 | hsa-mir-3653 | 1.36 | 0.02441 |
| 20538115 | SNORD125 | 1.36 | 0.02441 |
| 20532661 | ACA36B | 1.35 | 0.026362 |
| 20534102 | ENSG00000253049 | 1.35 | 0.033852 |
| 20538314 | hsa-mir-3133 | 1.35 | 0.019665 |
| 20532592 | 14qII-20 | 1.34 | 0.03405 |
| 20534594 | hsa-mir-135a-2 | 1.34 | 0.001689 |
| 20517898 | hsa-miR-3646 | 1.34 | 0.02918 |
| 20519582 | hsa-miR-4735-3p | 1.34 | 0.043167 |
| 20535170 | hsa-mir-523 | 1.34 | 0.013666 |
| 20532974 | ENSG00000206961 | 1.33 | 0.01268 |
| 20536553 | hsa-mir-3649 | 1.33 | 0.013443 |
| 20537634 | hsa-mir-6888 | 1.33 | 0.018356 |
| 20533697 | ENSG00000239059 | 1.32 | 0.011749 |
| 20500464 | hsa-miR-210-5p | 1.32 | 0.04854 |
| 20537477 | hsa-mir-6733 | 1.32 | 0.011878 |
| 20536465 | hsa-mir-4324 | 1.31 | 0.015402 |
| 20519502 | hsa-miR-4692 | 1.31 | 0.036857 |
| 20504550 | hsa-miR-758-3p | 1.31 | 0.018705 |
| 20533724 | ENSG00000239096 | 1.3 | 0.021019 |
| 20519645 | hsa-miR-4769-5p | 1.3 | 0.049823 |
| 20504551 | hsa-miR-1264 | 1.29 | 0.04065 |
| 20525726 | hsa-miR-6882-3p | 1.29 | 0.011229 |
| 20536281 | hsa-mir-1193 | 1.28 | 0.038512 |
| 20515564 | hsa-miR-3150a-5p | 1.28 | 0.009307 |
| 20525639 | hsa-miR-6838-5p | 1.28 | 0.002735 |
| 20519547 | hsa-miR-3529-3p | 1.27 | 0.007895 |
| 20535212 | hsa-mir-505 | 1.27 | 0.04128 |
| 20535201 | hsa-mir-519a-2 | 1.27 | 0.002957 |
| 20533968 | ENSG00000252459 | 1.26 | 0.008476 |
| 20537625 | hsa-mir-6879 | 1.26 | 0.023931 |
| 20533696 | ENSG00000239059 | 1.25 | 0.010802 |
| 20505963 | hsa-miR-922 | 1.23 | 0.020787 |
| 20532693 | ACA56 | 1.21 | 0.015673 |
| 20533634 | ENSG00000238972 | 1.21 | 0.019704 |
| 20533635 | ENSG00000238972 | 1.21 | 0.019704 |
| 20534200 | ENSG00000268874 | 1.21 | 0.015673 |
| 20503794 | hsa-miR-146b-3p | 1.21 | 0.04624 |
| 20517725 | hsa-miR-4268 | 1.21 | 0.012417 |
| 20536460 | hsa-mir-4320 | 1.21 | 0.047606 |
| 20518924 | hsa-miR-4524a-3p | 1.21 | 0.040453 |
| 20533942 | ENSG00000252356 | -1.21 | 0.037296 |
| 20534009 | ENSG00000252668 | -1.21 | 0.037615 |
| 20536228 | hsa-mir-3139 | -1.21 | 0.049231 |
| 20537197 | hsa-mir-5682 | -1.21 | 0.00177 |
| 20537244 | hsa-mir-6071 | -1.21 | 0.01793 |
| 20533336 | ENSG00000238515 | -1.22 | 0.022395 |
| 20536700 | hsa-mir-4446 | -1.22 | 0.04496 |
| 20525524 | hsa-miR-6781-3p | -1.22 | 0.00029 |
| 20534012 | ENSG00000252672 | -1.23 | 0.04211 |
| 20534131 | ENSG00000263442 | -1.23 | 0.000273 |
| 20534152 | ENSG00000264591 | -1.23 | 0.000273 |
| 20534159 | ENSG00000265325 | -1.23 | 0.000273 |
| 20534163 | ENSG00000265607 | -1.23 | 0.000273 |
| 20534183 | ENSG00000266646 | -1.23 | 0.000273 |
| 20534187 | ENSG00000266755 | -1.23 | 0.000273 |
| 20534367 | hsa-mir-24-2 | -1.23 | 0.033696 |
| 20515583 | hsa-miR-3160-3p | -1.23 | 0.03976 |
| 20538261 | U84 | -1.23 | 0.000273 |
| 20533066 | ENSG00000212342 | -1.24 | 0.049397 |
| 20533238 | ENSG00000238348 | -1.24 | 0.029372 |
| 20533952 | ENSG00000252405 | -1.25 | 0.020897 |
| 20500188 | hsa-miR-29b-3p | -1.25 | 0.025483 |
| 20517923 | hsa-miR-3668 | -1.25 | 0.036805 |
| 20519418 | hsa-miR-4640-3p | -1.25 | 0.017094 |
| 20519563 | hsa-miR-4724-3p | -1.25 | 0.006712 |
| 20534302 | HBII-85-11 | -1.27 | 0.043425 |
| 20534600 | hsa-mir-142 | -1.27 | 0.038726 |
| 20519646 | hsa-miR-4769-3p | -1.27 | 0.035332 |
| 20504356 | hsa-miR-615-5p | -1.27 | 0.03615 |
| 20533170 | ENSG00000221639 | -1.28 | 0.010151 |
| 20535219 | hsa-mir-510 | -1.28 | 0.007292 |
| 20533823 | ENSG00000251878 | -1.29 | 0.032177 |
| 20504217 | hsa-miR-487b-5p | -1.29 | 0.020397 |
| 20535139 | hsa-mir-489 | -1.29 | 0.03363 |
| 20519450 | hsa-miR-4662a-3p | -1.3 | 0.025994 |
| 20522016 | hsa-miR-5684 | -1.31 | 0.041153 |
| 20538224 | U68 | -1.31 | 0.028337 |
| 20533483 | ENSG00000238734 | -1.32 | 0.047946 |
| 20506866 | hsa-miR-1269a | -1.32 | 0.010259 |
| 20501036 | hsa-miR-200c-3p | -1.32 | 0.003315 |
| 20536285 | hsa-mir-3174 | -1.32 | 0.046908 |
| 20536296 | hsa-mir-548w | -1.32 | 0.000172 |
| 20534407 | hsa-mir-16-2 | -1.34 | 0.027007 |
| 20535305 | hsa-mir-567 | -1.34 | 0.013527 |
| 20529141 | hsa-miR-7849-3p | -1.34 | 0.047054 |
| 20506782 | hsa-miR-1233-3p | -1.35 | 0.002096 |
| 20533531 | ENSG00000238805 | -1.36 | 0.010571 |
| 20535879 | hsa-mir-1263 | -1.36 | 0.049957 |
| 20501198 | hsa-miR-361-3p | -1.36 | 0.02078 |
| 20537160 | hsa-mir-5585 | -1.36 | 0.005258 |
| 20533344 | ENSG00000238528 | -1.37 | 0.048146 |
| 20535849 | hsa-mir-548f-1 | -1.37 | 0.035514 |
| 20519466 | hsa-miR-219b-3p | -1.38 | 0.001075 |
| 20537214 | hsa-mir-4666b | -1.39 | 0.048061 |
| 20500476 | hsa-miR-216a-3p | -1.4 | 0.007189 |
| 20517999 | hsa-miR-3713 | -1.4 | 0.000004 |
| 20537629 | hsa-mir-6883 | -1.4 | 0.02155 |
| 20536996 | hsa-mir-499b | -1.41 | 0.03678 |
| 20525419 | hsa-miR-6728-3p | -1.41 | 0.001799 |
| 20521842 | hsa-miR-548av-5p | -1.42 | 0.002566 |
| 20533881 | ENSG00000252129 | -1.44 | 0.020974 |
| 20533933 | ENSG00000252305 | -1.44 | 0.020974 |
| 20534846 | hsa-mir-369 | -1.44 | 0.027845 |
| 20504555 | hsa-miR-668-3p | -1.46 | 0.023893 |
| 20535307 | hsa-mir-551b | -1.5 | 0.044882 |
| 20500746 | hsa-miR-140-3p | -1.54 | 0.026188 |
| 20506799 | hsa-miR-1205 | -1.55 | 0.004107 |
| 20537913 | hsa-mir-7515 | -1.56 | 0.013157 |
| 20533218 | ENSG00000238327 | -1.57 | 0.021854 |
| 20534541 | hsa-mir-224 | -1.61 | 0.006371 |
| 20518918 | hsa-miR-4520a-3p | -1.62 | 0.028431 |
| 20519616 | hsa-miR-371b-3p | -1.64 | 0.039012 |
| 20501286 | hsa-miR-151a-5p | -1.67 | 0.019646 |
| 20538161 | U31 | -1.68 | 0.048578 |
| 20501284 | hsa-miR-323a-3p | -1.69 | 0.024224 |
| 20525665 | hsa-miR-6852-5p | -1.71 | 0.005739 |
| 20503803 | hsa-miR-494-3p | -2.38 | 0.046903 |

**Supplementary Table 3.** List of pathways that are significantly perturbed by miRNAs secreted through ADEVs by C9-ALS iAstrocytes versus controls when applying a cutoff of p≥0.05 and fold change ≥1.5 or ≥1.2. The p values were obtained through DAVID gene enrichment analysis.

| **Pathways** | **FC>1.5**  **pvalue** | **FC>1.2**  **pvalue** |
| --- | --- | --- |
| hsa04360:Axon guidance | 5.94E-04 | 2.44E-07 |
| hsa04350:TGF-beta signaling pathway | 1.86E-05 | 2.48E-07 |
| hsa04520:Adherens junction | 5.32E-04 | 1.19E-06 |
| hsa04144:Endocytosis | 5.75E-04 | 1.25E-05 |
| hsa04810:Regulation of actin cytoskeleton | 0.0122 | 2.90E-04 |
